# Leadership shift in the global soybean market: Dynamic connectedness approach (TVP-VAR)

**DOI:** 10.1016/j.heliyon.2024.e36071

**Published:** 2024-08-09

**Authors:** Gustavo María Barboza Martignone, Bikramaditya Ghosh, Karl Behrendt, Dimitrios Papadas

**Affiliations:** a FLAM Department, Harper Adams University, Newport, TF10 8NB, UK; b Symbiosis Institute of Business Management, Symbiosis International, Deemed University, Bengaluru, 560100, India; c AI Deep Economics, Department of Applied Economics, Uruguay

**Keywords:** Price transmission, International soybean market, Connectedness index, TVP-VAR, Risk of spillover, Connectedness approach, AI Deep Economics

## Abstract

The price transmission in international soybean market has been extensively examined. However, recent econometric advancements have enabled the application of dynamic connectedness methodology as outlined by Antonakakis and Gabauer (2017) [1], which is based on a Time-varying Parameter Vector Autoregressive (TVP-VAR) model. This approach captures the time-varying connectedness of time series, considering potential risk shock emitters and receivers. The connectedness index (Diebold and Yilmaz, Jan. 2012) [2] was developed using Generalized Forecast Error Variance Decomposition (GFEVD) and the Generalized Impulse Response Function (GIRF) (Koop et al., Sep. 1996; Pesaran and Shin, Jan. 1998) [3,4]. This study aims to understand the dynamic connectedness and price leadership. The research examined markets including the US Soybean futures market (Chicago Board of Trade), Rotterdam Port spot market representing the European soybean market, Paranaguá port representing Brazil, Argentina represented by Rosario Futures and Spot, and the Chinese domestic spot market and the Dalian futures on behalf of China. The research spanned approximately ten years, from September 2009 to May 2019. The findings suggest that the soybean market has reached a high level of maturity, able to withstand exogenous shocks for at least the past seven years. The net pairwise directional connectedness revealed dynamic and bidirectional causality. The dynamic connectedness index showed a highly connected and developed market in the West (Chicago Futures, Rotterdam, Paranaguá, and Rosario Futures and Spot). However, the connectedness between Western and Eastern markets was relatively low, indicating some level of market isolation. Furthermore, the pairwise connectedness index between Eastern market (China spot and Dalian Futures) was also considerable low. Lastly, Paranaguá and Rosario overtook Rotterdam as price-leading markets and were identified as the primary net transmitters of shocks, indirectly implying causality. Chicago, Rosario, and Paranaguá formed a triumvirate leading international prices, while Rotterdam adopted a secondary leading position, deviating from its historical role as the price leader.

## 1 Introduction

In the complex and interconnected world of soybeans markets, understanding the dynamics of market integration and price transmission becomes crucial for economists and policymakers alike. This research embarks on an innovative journey to unravel the intricacies of soybean markets integration by exploring beyond the traditional realm of Johansen and Engle-Granger cointegration approaches, which have been the foundation for previous studies in diagnosing the interlinkages among various markets. Despite the invaluable insights provided by these cointegration methodologies, which primarily focus on static relationships, there exists an increasing need to delve into the dynamic aspects of the different soybean markets connectedness in dynamics terms. This research aims to bridge this gap by concentrating on the dynamic connectedness [1,2] a concept that transcends the static nature of cointegration, thereby offering a more time dynamic understanding of price time-varying behaviours and spillover mechanisms. By conducting a comprehensive examination of a diverse array of markets within the horizontal or spatial dimension, this research encompasses pivotal trading hubs such as the U.S. Soybean Futures market (Chicago Board of Trade), the Rotterdam port spot market an essential nexus for the European soybean trade and the Paranaguá port spot market, which is representative of the Brazilian soybean sector. Additionally, it incorporates the Argentinian futures and spot markets of Rosario, the Chinese domestic spot market (Heihe Grain Bureau), and the Dalian Commodity Exchange (Dalian soybean futures). This study provides an extensive analysis spanning approximately a decade, from September 2009 to May 2019, thereby offering robust insights into the temporal dynamics and inter-market linkages of these critical global soybean markets.

The international soybean market, known for its efficiency and integration [3], serves as a fertile field for this study. With a rich history of research delving into various economic phenomena ranging from price transmission to market power, the soybean market exemplifies a well-studied yet continually evolving domain. This research, therefore, seeks to add a fresh perspective by employing a time-varying parameter methodology [1] to capture the dynamic connectedness or spillover process and price leadership in a globalized context marked by incessant exogenous shocks and market condition changes.

The methodology section sets the stage for a comprehensive exploration of the Time-Varying Parameter Vector Autoregression (TVP-VAR) and the connectedness index [2]. This methodology [1] allows us to improve the comprehension of the mechanism of price time-varying and price spillover. As the narrative unfolds, the results are systematically presented, juxtaposed with previous empirical findings, to shed light on the causal relationships and capturing exogenous shocks. This comparative analysis not only enriches our understanding of market dynamics but also highlights the limitations and strengths of the chosen methodological approach.

Following the discussion and policy implications section, this research endeavours to pile together the threads of findings, historical contexts, and contemporary policy landscapes. By synthesizing insights from the study with prior research, it aims to offer cogent interpretations of the results and their broader implications. Furthermore, the discussion extends to future expectations and market trajectories, providing a forward-looking view of the international soybean market and its constituents.

### 1.1 Key concepts

Price Transmission (PT) and Asymmetric Price Transmission (APT) explore the dynamics of price changes within market levels and their unequal propagation respectively. PT emphasizes how market-level price alterations influence other market levels, essential for market efficiency by indicating supply and demand shifts [4]. APT indicates market inefficiencies where price increases are transmitted differently compared to price decreases, often due to market power or cost structures [5]. The Connectedness Index or Risk of Spillover [2] measures the impact of market disturbances on others, crucial in finance for understanding asset or market volatility propagation [6]. A higher index signifies greater market interdependence, suggesting that shocks in one area can significantly impact others [7]. Cointegration, a statistical concept in econometrics, assesses long-term relationships among time series variables. If variables are non-stationary but their combination is stationary, they are considered cointegrated, suggesting a stable long-term equilibrium between them despite short-term fluctuations. This is vital for analysing economic relationships, such as between prices of substitutes or interest rates and investment [8].

### 1.2 Previous research and empirical work

The international soybean market is known for being a highly efficient and integrated market, making it a fertile field for applied economics. Many researchers over the years have focused on this market, studying different economic phenomena such as price transmission (PT) [3,9], price formation [10], price volatility [11], seasonal PT [12], elasticity of PT [13], asymmetric PT (APT) [14,15], effects of timing of crop and trade [16], market integration and market power [17] and cross price transmission [18]. Most of these researchers have used classical cointegration, AR models, and price transmission methodologies, which are useful tools that test economic theory against observed reality.

[2,6] attempted to study the dynamics of the main players from the supply and demand side using a cointegration and price transmission methodology. However, this methodology has several limitations. For example, the availability and quality of data can lead to inaccurate or unreliable results. The choice of model specification can also affect the results of price transmission analysis, as different models may yield different results. Additionally, market structure, such as the degree of competition and market power, can also affect the results of price transmission analysis. Furthermore, many price transmission models assume that parameters are constant over time, but they may vary. This can lead to biased or unreliable results if not accounted for [19].

This research builds on previous research that has already analysed the same data set; therefore, it is necessary to understand the previous results to contrast the different methodology results. Previous researchers had transformed the data into natural logarithms and first differences in order to make the data stationary. The Augmented Dickey-Fuller (ADF) test and ADF test with breaks were performed by Ref. [3] concluding that the data was stationary in the first difference I(1). Later on [3], performed Phillips-Perron unit root test, confirming the order of integration I(1).

The previously mentioned authors concluded that all markets were cointegrated to different degrees, using the Johansen cointegration test and, after correction by structural breaks, all markets presented cointegration among and between them. Using the Engle-Granger cointegration test [3], struggled to find cointegration among all the time series, especially among and between series that presented structural breaks correlated with government intervention. The China spot market was the least cointegrated series, only presenting cointegration equations with Rosario Spot and Dalian Futures. The authors suggested the lack of statistical power of the Engle-Granger test, with the extenuating effect of structural government intervention that has generated several structural breaks, fading the cointegration vectors. The cointegration under asymmetry test by Ref. [20] applied under TAR and MTAR models by Ref. [15] showed that the model results presented no trace of asymmetric price transmissions among the markets, but the cointegration test under asymmetry [20] showed that it was always necessary to correct for dummy variables (structural breaks) in order to show full cointegration. The TAR model for China showed a lack of cointegration under asymmetry for Rotterdam, Paranaguá, and Rosario (China is only cointegrated with Dalian futures). The cointegration test under asymmetry [20] for the MTAR model showed that all series are cointegrated after being corrected for structural breaks. This opens the question of how integrated the markets are. Since cointegration is a static concept, it cannot be totally extrapolated to dynamic markets. Therefore, it is necessary to adopt another approach.

Several researchers have utilized the dynamic connectedness methodology, based either on a Time-Varying Parameter in a Vector Autoregressive model (TVP-VAR) or on a Quartile Regression model (QVAR) [1]. studied the uncertainty transmission between developed economies using these methodologies. They examined the spillover effect among the EU, US, Japan, the UK, and Canada, and their findings suggested significant transmission of uncertainty from the EU to the US [21]. studied the connectedness among crude oil, grains, livestock, sugar, soybean oil, cocoa, corn, lean hogs, soybeans, wheat, and cattle using the connectedness methodology based on a TVP-VAR. The authors found high net connectedness among the studied sample 70 % implying strong cointegration or co-movement among the selected commodities. The results suggested that crude oil most significantly affects other markets. However, it is also affected by changes in agricultural commodities markets, shifting from being a net transmitter to a net receiver over time. Only livestock and grain displayed consistent net transmitter behaviour over time.

[22] used the dynamic connectedness methodology based on a Quartile Regression model (QVAR) to study the risk of spillover, or connectedness, for fourteen agricultural commodities: poultry, beef, soybean, coffee grains, cocoa, palm oil, corn, wheat, tea, groundnut oil, palm oil, sugar, orange juice, and rice. Their results indicated a high connectedness index over 55 % for the entire period from 1965 to 2022. This suggests that the risk of spillover hasn't decreased in recent years, and the agricultural commodity markets are quite susceptible to external shocks. They also found that soybean, corn, wheat, and palm oil are net transmitters, leading in prices and transmitting the most to other commodities.

## 2 Materials and methods

The descriptive results for the selected dataset showed low variance in the data and a Gaussian nature of the distribution. The majority of the data was grouped under the mean, meaning that a standard quantile regression could not express the nature of the time series. Therefore, a Time-varying parameter Vector Autoregressive (TVP-VAR) model was built. This type of model enables us to capture the time-varying nature of the time series. The TVP-VAR model assumes that the data is stationary. To achieve this, the data was mean-reverted, assuming that the data converges to the average price over time, transformed into natural logarithm twice in order to achieve homeostasis and approximate the data under normality. This will make the series stationary and facilitate the prediction mechanism to perform better. To check if the transformed data met the previously mentioned assumptions, the Jaque-Bera test for normality and the Elliot, Rothenberg, and Stock (ERS) test for stationarity were applied. After the data met the correct specification, the TVP-VAR model was built using the [1] method. From the vector moving average, the time-varying coefficient was extracted, a key piece of the connectedness [2], used in the Generalized Forecast Error Variance Decomposition (GFEVD) [23,24] and the Generalized Impulse Response Function (GIRF).

The GFEVD and the GIRF are crucial tools in the connectedness approach, which is employed to analyze the interdependence and dynamic interactions among multiple time series variables. The GFEVD is utilized to understand the proportion of the forecast error variance of a particular variable that can be attributed to shocks in other variables within the system. Unlike the traditional variance decomposition, which requires orthogonal shocks and is sensitive to the ordering of variables, the GFEVD allows for the decomposition of forecast error variances using shocks that are not orthogonal. This makes the GFEVD a more flexible and robust tool, especially in systems where variables are contemporaneously correlated. By decomposing the forecast error variance, the GFEVD provides a detailed picture of how shocks propagate through the system over time, highlighting the relative importance of each variable in influencing others.

The GIRF measures the response of each variable in the system to a shock in any one variable, considering the generalized nature of the shocks. This function extends the traditional impulse response analysis by not requiring orthogonal shocks, thus making the analysis invariant to the ordering of variables in the vector autoregression (VAR) model. The GIRF traces out the effect of a one-time shock to one of the innovations on the current and future values of the endogenous variables, offering a more accurate depiction of the dynamic interrelationships among variables. In the connectedness approach, these tools are integral for quantifying and visualizing the interconnections among variables. The GFEVD constructs connectedness tables, summarizing the degree of connectedness among all pairs of variables, while the GIRF provides a temporal dimension to this analysis, examining how shocks to one variable affect the others over time. Together, these tools enhance the connectedness approach by offering a comprehensive framework for analyzing interdependencies and dynamic interactions in a multivariate time series context, thereby uncovering the underlying structure of market dynamics and informing risk management practices and policymaking.

### 2.1 Methodological limitations

Despite the robustness and flexibility of the Time-Varying Parameter Vector Autoregressive (TVP-VAR) model in capturing the dynamic nature of time series data, several limitations must be acknowledged. Firstly, the assumption of stationarity is critical for the TVP-VAR model. Achieving stationarity required transforming the data through mean-reversion and double natural logarithm transformations, which, while effective in this context, may not be universally applicable or appropriate for all datasets. This transformation process also assumes that the data will converge to an average price over time, which may not hold true in markets with structural breaks or non-stationary behaviours.

Additionally, the methodology relies heavily on the validity of the Jaque-Bera test for normality and the Elliot, Rothenberg, and Stock (ERS) test for stationarity. These tests, while standard, may not fully capture the complexities or subtle deviations from the assumptions in real-world data. Another limitation arises from the use of the Generalized Forecast Error Variance Decomposition (GFEVD) and Generalized Impulse Response Function (GIRF). Although these tools provide a detailed picture of shock propagation and dynamic interrelationships, their effectiveness can be compromised in systems with high dimensionality or where the relationships between variables are not linear. Furthermore, the results from the GFEVD and GIRF can be sensitive to the model specification and the quality of the data. Hence, the findings should be interpreted with caution, particularly in cases where the underlying data may exhibit significant noise or outliers that were not fully addressed during the preprocessing stages. These limitations highlight the need for careful application and interpretation of the TVP-VAR model and its associated tools in the connectedness approach.

### 2.2 Averaging data pricing

This involves calculating the mean (average) price of the data over a specified period. Helps to smooth out short-term fluctuations and highlight longer-term trends in the data.

Mathematical Representation (equation (1)):

(1) $$\bar{X}=\frac{1}{N}\sum_{i=1}^{N}X_{i}$$

where $\bar{X}$ is the average price, N is the number of observations, and $X_{i}$ represents the individual data points.

### 2.3 Mean reversion

Mean reversion is a statistical property of a time series where values tend to return to the mean (average) level over time. In finance, mean reversion suggests that high prices will tend to decrease and low prices will tend to increase over time, converging to the average price.

For instance (equation (2)):

(2) $$\text{For}\;a\;\text{time}\;\text{series}\;X_{t+1}=\mu +\theta \left(X_{t}‐\mu \right)+\epsilon_{t}$$

Where:

μ is the long-term level

θ is the speed of reversion to the mean (0 < θ < 1).

$\epsilon_{t}$ is a random error term.

### 2.4 Transformation into natural logarithm

This transformation aims to achieve data homeostasis (stability) and normalizing the data distribution.

If $X_{t}$ represents the original time series, then the transformed series $Y_{t}$ can be represented as equation (3).

(3) $$Y_{t}=\ln\;\left(X_{t}\right)$$

where ln is the natural logarithm function.

### 2.5 TVP-VAR

As before mentioned, TVP-VAR method of [1] to examine how transmission changes over time was applied. This approach builds on the original connectedness method of [2,25] by incorporating a Kalman Filter estimation with forgetting factors to account for varying variances. The TVP-VAR model can be represented by the following equation (4)(5).

(4) $$Yt=\beta_{t}z_{t-1}+\epsilon_{t}\;\epsilon_{t}|F_{t-1}∼N\left(0,S_{t}\right)$$

(5) $$vec\left(\beta_{t}\right)=vec\left(\beta_{t-1}\right)+\nu_{t}\;\nu_{t}|F_{t-1}∼N\left(0,R_{t}\right)$$

The vectors $y_{t-1}$ and $z_{t-1}$ = [$y_{t-1}$, …, $y_{t-p}$]′ represent N × 1 and $N_{p}$ × 1 dimensional vectors, respectively. The time-varying coefficient matrix is represented by $\beta_{t}$, which is an N × N p dimensional matrix. The error disturbance vector is represented by $\epsilon_{t}$, which has an N × 1 dimensional with an N × N time-varying variance-covariance matrix, $S_{t}\text{.}$ $vec\left(\beta_{t}\right)$, $vec\left(\beta_{t-1}\right)$ and $\nu_{t}$ are N^2^ p × 1 dimensional vectors and $R_{t}$ is an N^2^ p × N^2^ p dimensional matrix. In order to calculate the generalized impulse response functions (GIRF) and generalized forecast error variance decomposition (GFEVD) [23,24]**,** the VAR was transformed into its vector moving average (VMA) representation (equations (6), (7))).

(6) $$Yt=\sum_{j=0}^{\infty }L^{\prime }W_{t}^{j}L\epsilon_{t-j}$$

(7) $$Yt=\sum_{j=0}^{\infty }A_{jt}\epsilon_{t-j}$$

L and W are dimensional matrix (Equations (9), (8)))

(8) $$W=\left[\beta_{t};I_{N\left(p-1\right)},0_{N\left(p-1\right)\times N}\right]\;\left(N_{p}\times N_{p}\right)$$

(9) $$L={\left[I_{N,‥,}0_{p}\right]}^{\prime }\;\left(N_{p}\times N\right)$$

And $A_{jt}$ is an N×N dimensional matrix

The GIRFs, or Generalized Impulse Response Functions, show how all variables react when there is a shock in variable j. Since there is no structural model available, the team compares a forecast for J steps ahead where variable j is shocked to one where it is not shocked. The difference between the two is attributed to the shock in variable j and can be determined through the equation provided (10).

(10) $$GIRF_{t}\left(J,\delta_{j,t},F_{t-1}\right)=E\left(\epsilon_{j,t}=\delta_{j,t},F_{t-1}\right)-E\left(\;Y_{t+1}|F_{t-1\;}\;\right)$$

(11) $$\psi_{j,t}^{g}\left(J\right)=\frac{A_{J,t}S_{t}\epsilon_{j,t}}{\sqrt{S_{jj,t}}}+\frac{\delta_{j,t}}{\sqrt{S_{jj,t}}}\;\delta_{j,t}=\sqrt{S_{jj,t}}$$

(12) $$\psi_{j,t}^{g}\left(J\right)=S_{jj,t}^{-\frac{1}{2}}A_{J,t}S_{t}\epsilon_{j,t}$$

The GIRFs for the variable j is represented by $\psi_{j,t}^{g}\left(J\right)$ where J represents the forecast horizon (equations (11), (12))). The selection vector represented by $\delta_{j,t}$ with zero or one on the j th position. And $F_{t-1}$ set until t −1. Subsequently, GFEVD is calculated interpreted as the variance portion one variable has in others (equation (13)).

(13) $${\tilde{\phi }}_{ij,t}^{g}\left(J\right)=\frac{\sum_{t=1}^{J-1}\psi_{ij,t}^{2,g}}{\sum_{j=1}^{N}\sum_{t=1}^{J-1}\psi_{ij,t}^{2,g}}$$

Assuming the following (equations (14), (15))):

(14) $$\sum_{j=1\;}^{N}{\tilde{\phi }}_{ij,t}^{g}\left(J\right)=1$$

(15) $$\sum_{i,j=1,i\neq j}^{N}{\tilde{\phi }}_{ij,t}^{g}\left(J\right)=N$$

Afterwards is possible construct the total connectedness index (TCI) following (equation (16)):

(16) $$C_{t}^{g}\left(J\right)=\frac{\sum_{i,j=1,i\neq j}^{N}{\tilde{\phi }}_{ij,t}^{g}\left(J\right)}{\sum_{i,j=1}^{N}{\tilde{\phi }}_{ij,t}^{g}\left(J\right)}\;*100=\frac{\sum_{i,j=1,i\neq j}^{N}{\tilde{\phi }}_{ij,t}^{g}\left(J\right)}{N}\;*100$$

This connectedness method demonstrates how a shock in one variable affects other variables. We first consider the scenario where a shock in variable i is transmitted to all other variables j, known as total directional connectedness to others, as defined in the following equation (17).

(17) $$C_{i\to j,t}^{g}\left(J\right)=\frac{\sum_{j=1,i\neq j}^{N}{\tilde{\phi }}_{ji,t}^{g}\left(J\right)}{\sum_{j=1}^{N}{\tilde{\phi }}_{ji,t}^{g}\left(J\right)}\;*100$$

Next, we determine the directional connectedness that variable i receives from variables j, referred to as total directional connectedness from others, which is defined as equation (18).

(18) $$C_{i\leftarrow j,t}^{g}\left(J\right)=\frac{\sum_{j=1,i\neq j}^{N}{\tilde{\phi }}_{ij,t}^{g}\left(J\right)}{\sum_{i=1}^{N}{\tilde{\phi }}_{ij,t}^{g}\left(J\right)}\;*100$$

Finally, the net total directional connectedness was calculated by subtracting the total directional connectedness to others from the total directional connectedness from others (equation (19)).

(19) $$C_{i,t}^{g}=C_{i\to j,t}^{g}\left(J\right)-\;C_{i\leftarrow j,t}^{g}\left(J\right)$$

The net total connectedness sign shows if variable i is driving the network or being driven by the network. Where if $C_{i,t}^{g}\;>0$ , i is driving the network or if $C_{i,t}^{g}\;<0$ , i is being driven by the network. Finally, we analyze the net total directional connectedness by computing the net pairwise directional connectedness (NPDC) to examine the bidirectional relationships (equation (20)).

(20) $$NPDC_{ij}\left(J\right)=\frac{{{\tilde{\phi }}_{ji,t}^{g}\left(J\right)-\;\tilde{\phi }}_{ij,t}^{g}\left(J\right)}{N}\;*100$$

### 2.6 Connectedness decomposition

Since we are studying the spillovers between two countries, we are interested in how much of the spillovers is transmitted within the country and how much is transmitted from one country to another. The decomposition of k countries can be represented as follows (equation (21)):

(21) $$\phi \left(J\right)={\left[{\tilde{\phi }}^{g}\right]}_{ij,t}\left(J\right)=\left[C_{11}\;C_{12}\cdots C_{1k}\;C_{21}\;C_{22}\cdots C_{2k}\vdots \vdots \ddots \vdots C_{k1}\;C_{k2}\cdots C_{kk}\;\right]$$

$C_{ii}$ encompassed the internal spillover of country i.

$C_{ij}$ represents the spillover of country j to country *i*

Next, to determine the internal and external spillovers, it requires to set $diag\left(C_{ii}\right)=0$ and determine:

(22) $$TO_{ij}=\sum_{n=1}^{k}C_{ij,nm}$$

$TO_{ij}$ stands for the total country-specific connectedness to others (equation (22)).

(23) $${FROM}_{ij}=\sum_{m=1}^{k}C_{ji,nm}$$

${FROM}_{ij}$ refers to the total country-specific connectedness from others (equation (23)).

(24) $${NET}_{ij}=TO_{ij}-FROM_{ij}$$

${NET}_{ij}$ represents the net total country-specific connectedness. (equation (24))

(25) $$NI_{ij}=\sum_{n=1}^{k}\sum_{m=1}^{k}C_{ij,nm}-\sum_{n=1}^{k}\sum_{m=1}^{k}C_{ji,nm}$$

$NI_{ij}$ represents the net international total market-specific connectedness (equation (25)).

This equation (22) is arrived at by recognizing that $TO_{ij}$ is a summation over *nm* subscript where *i* is the first subscript, while ${FROM}_{ij}$ is a summation over the *nm* subscript where *i* is the second subscript. The difference between these two sums (i.e., $TO_{ij}-{FROM}_{ij}$ *T*) gives you $NI_{ij}$, which represents the net balance of connectedness for market *i* in relation to country *j*, considering all sectors.

## 3 Results

### 3.1 Descriptive statistics and diagnostic test

**Chart 1:**
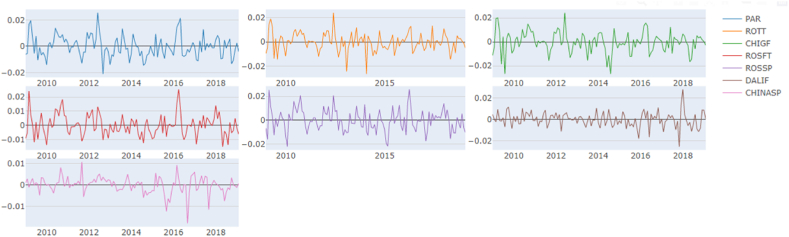
Data as a time series.

In Chart 1, we can observe all the time series after they have been double-logged and mean-reverted. The descriptive statistics are presented in Table 1. As the data has been double logged, the mean and variance are zero, which improves the model's robustness and allows for modelling under the Gaussian assumption of zero mean and unit variance. The kurtosis levels for nearly all time series are relatively low (platykurtic), except for the China Spot market, which has a kurtosis of 4.2. This higher kurtosis makes it leptokurtic and exhibits a fat tail (distribution skewed towards the tail). The elevated kurtosis of the China Spot market can be partially attributed to structural market interventions. The Elliott, Rothenberg, and Stock (ERS) test indicated that all-time series are stationary following the aforementioned transformation.

**Table 1:** Descriptive statistics.

	PAR	ROTT	CHIGF	ROSFT	ROSSP	DALIF	CHINASP
Mean	0	0	0	0	0	0	0
Variance	0	0	0	0	0	0	0
Skewness	0.442**	−0.186	−0.308	0.644***	0.226	−0.032	−1.020***
	−0.043	−0.379	−0.151	−0.005	−0.287	−0.877	0
Kurtosis	0.368	1.394**	0.948*	0.870*	0.239	2.466***	4.195***
	−0.287	−0.014	−0.051	−0.065	−0.413	−0.001	0
JB	4.663*	10.583***	6.492**	12.266***	1.33	30.935***	110.627***
	−0.097	−0.005	−0.039	−0.002	−0.514	0	0
ERS	−3.509***	−2.639***	−2.726***	−2.443**	−3.624***	−3.582***	−4.910***
	−0.001	−0.009	−0.007	−0.016	0	−0.001	0
Q (10)	20.879***	8.582	16.805***	19.673***	14.735***	7.959	17.445***
	0	−0.132	−0.002	0	−0.006	−0.173	−0.001
Q2(10)	7.444	2.354	10.147*	5.289	7.923	15.334***	2.759
	−0.213	−0.902	−0.065	−0.463	−0.175	−0.005	−0.853

### 3.2 Average and dynamic total connectedness measures

**Chart 2:**
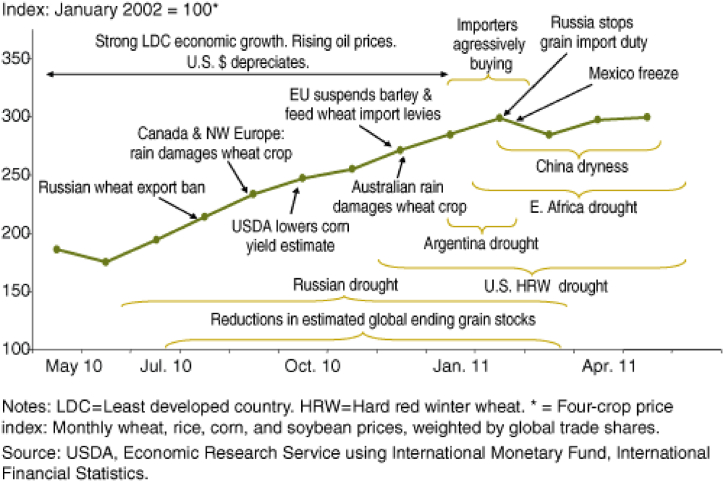
Crop index over the time.

**Chart 3:**
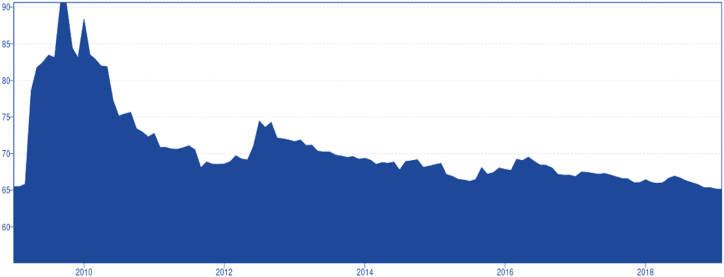
Total Connectedness Index over the years.

In Chart 2, the Total Connectedness Index (TCI) or the Total Spillover Index (TSI) can be observed over the years. Between mid-2010 and mid-2011, there was a noticeable increase from 65 to 90, indicating a crisis or disturbance in the market during that period. This can be attributed to large and rapid price increases followed by larger swings in prices that occurred during 2010 and 2011, which were caused by various exogenous factors [26]. Some factors directly affected soybean supply. In November 2010, the “La Niña" meteorological phenomenon of high temperatures combined with a lack of rain generated a significant drought across Argentina, considerably reducing soybean prospects. Increased demand for meat, beef, and pork caused the price of feed, including soybean, to rise. Additionally, importers began adopting aggressive strategies to ensure supply by contracting grain quantities for the following 4–6 months [26]. Many factors added uncertainty and affected other agricultural commodities. Due to the cross-commodity price transmission or volatility spillovers among different energy commodities (such as oil prices to agricultural prices), these changes might have influenced soybean prices. The strong economic growth experienced by less developed countries and the depreciation of the US dollar put inflationary pressure on food and grain prices. Alongside many other factors, this created uncertainty in market price swings and spillover effects across different agricultural commodities (Chart 3).

Source [26].

By 2012, the incidence of the disturbance has disappeared, however, around March to May there was another turbulence in the market, peaking the index to 75. After 2014, the index stabilized around 67 until the last period recorded, this clearly indicates a mature and steady market capable of overcoming market shocks or distortions. This follows the case of the US/China Trade war, as mentioned by Ref. [3] where the authors suggested that the international market of soybeans was capable of arbitrage around tariffs and circumvent them, rearranging the trade flows in order to get around the trading barriers.

**Chart 4:**
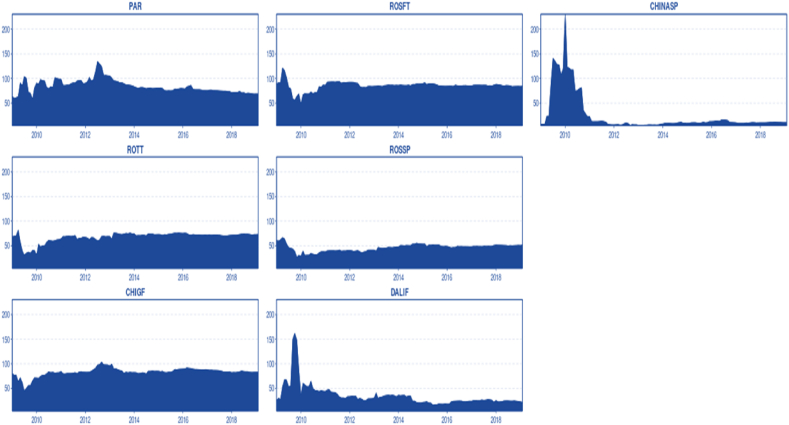
To others.

Moreover, this finding suggests a degree of market insulation from exogenous shocks, as the TCI index remains steady over time, at least since 2014. In other words, while agricultural commodity markets tend to be quite volatile, soybean prices exhibit less random fluctuation and appear relatively stagnant. This could mean that supply and demand are stable, or that the market is highly developed and capable of absorbing shocks. Both supply and demand have been growing steadily, driven by China's increasing soybean consumption and the consistent expansion of soybean farming areas in Brazil [27], and Argentina [28] which in turn boosts supply. As a result, it is plausible to propose that the market's level of development, efficiency, and integration are the primary factors explaining why the TCI index remains steady and relatively constant over time. The TCI hovers around 65 %, indicating a moderate spillover risk for almost a decade. In Chart 4, the transmitted shocks or spillover effects from the studied markets to other markets can be observed. Paranaguá, Rosario Futures, Chicago, and Rotterdam are the markets that transmit shocks most significantly (after mid-2012), as indicated by their higher connected net index. The spillover for these markets remains relatively stagnant over time. China, from 2009 to 2011, exhibited a high connectedness index, transmitting shocks to others. However, after 2011, this ceased, and the connectedness index sharply decreased and remained low, almost marginal, and steady for the last period, suggesting a market insulation process caused by government interventions. Dalian Futures exhibited similar behaviour, with the TCI peaking in mid-2009 and sharply decreasing until 2010. Since then, the connectedness index to others has remained low and relatively steady (excluding a series of minor shocks), without significant spillover or influence on other markets.

**Chart 5:**
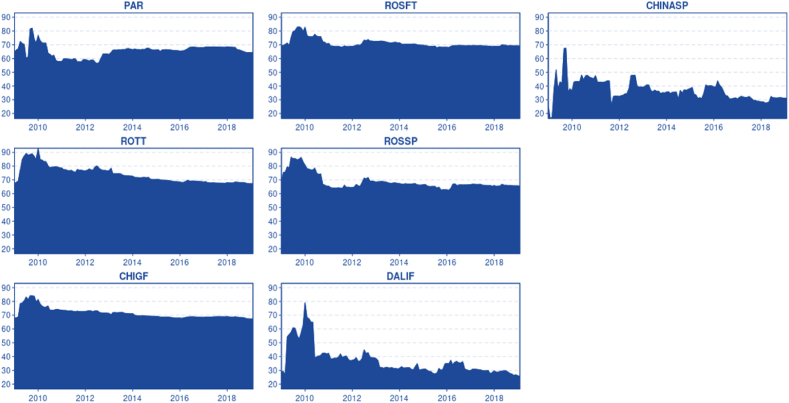
From others.

Chart 5 also displays the shocks or spillover received from other markets. Rotterdam, Rosario Spot, and China Spot are net receivers, meaning they tend to be more affected by other market shocks than influencing other markets. Overall, all markets tend to be highly influenced by external markets, which is normal, following the Law of One Price (LOOP) and market arbitrage processes. As previously mentioned, the China Spot market has a very low or marginal impact on other markets (Chart 2); however, the connectedness index from other markets tends to be significantly higher than the connectedness index to other markets. This indicates that the China domestic Spot market has limited importance on an international scale, exerting marginal influence on other markets, yet being influenced by international markets. It is important to understand that the Chinese government has protected its domestic market from the international market to ensure national food security. However, this market plays a minor role in meeting domestic demand. The domestic supply produces only 16 million tonnes, while 102 million tonnes of soybean are provided by the international market [29].

In Table 2, it is possible to observe a bidirectional influence of different markets. The connectedness of the system is divided into two forms. First, the last column named “FROM” shows the total aggregate amount of shocks that all the considered markets are receiving from the entire system. The estimation of this column's value is the result of adding all the horizontal numbers, apart from the on-diagonal numbers. Second, the last row “TO” expresses the transmission of shocks for each soybean market to the entire system. The value of this row is calculated by summing all the vertical numbers, excluding the on-diagonal numbers.

**Table 2:** Average dynamic connectedness table.

	PAR	ROTT	CHIGF	ROSFT	ROSSP	DALIF	CHINASP	FROM
PAR	34.49	13.56	19.11	16.68	7.68	4.48	3.99	65.51
ROTT	18.93	26.26	20.03	15.76	9.9	6.87	2.25	73.74
CHIGF	19.3	18.51	28.62	17.54	7.93	6	2.09	71.38
ROSFT	16.18	12.95	15.69	28.97	16.97	6.46	2.77	71.03
ROSSP	11.83	12.99	9.89	25.04	31.48	3.98	4.79	68.52
DALIF	8.33	4.96	7.55	5.52	2.09	63.29	8.25	36.71
CHINASP	9.19	4.39	10.59	4.41	1.15	6.98	63.28	36.72
TO	83.75	67.37	82.88	84.95	45.72	34.78	24.14	423.6
Inc.Own	118.25	93.63	111.5	113.92	77.21	98.08	87.42	TCI = 67 %

The Paranaguá spot market appears to have two thresholds of influence. The first threshold shows a strong influence on Chicago futures (19.3 %), Rotterdam (18.93 %), Rosario Futures (16.18 %), and is associated with a higher degree of market freedom, market integration, increased connectedness, and volatility spillover. This low-influence threshold follows a positive correlation with liberalization or a negative correlation with market intervention and market insulation.

The second threshold is a low-influence zone, where the Paranaguá market only influences Rosario Spot by around 11.83 %, China Spot by about 9.19 %, and Dalian Futures by 8.33 %. This low-influence threshold is associated with a high degree of market intervention, market insulation, and lower overall connectedness. Despite the average connectedness, the table shows bidirectional causality [3]. empirically demonstrated, using the Granger causality test, that there is no influence of the Paranaguá Spot on Chicago and Rotterdam, in contrast with the results of the Connectedness table (Table 2). It is possible to agree with the authors that the influence of the Paranaguá spot on the rest of the studied markets was noticeable in both investigations. The total spillover from the Paranaguá market transmitted to (“TO”) other markets is 83.75 %, making it the second-highest market to transmit shocks, and including itself, it is the highest transmitter. The Paranaguá market is influenced mostly by itself (34.49 %), followed by Chicago (19.11 %), Rosario Futures (16.68 %), Rotterdam (13.56 %), and marginally influenced by Rosario Spot, Dalian Futures, and China Spot (7.6 %, 4.48 %, and 3.99 % respectively). The influence of Rotterdam tends to be less than expected, and the influence of Rosario Futures is higher than Rotterdam on Paranaguá, which contrasts with [11],who proposed that Paranaguá and Rosario Futures were satellite markets of Rotterdam. Finally, we can affirm that Paranaguá is a net transmitter, transmitting shocks at 83.75 % “TO” and receiving shocks “FROM” at 65.51 %, giving Paranaguá a net transmission of spillovers of 18.24 %.

In Tables 2 and it appears that the Rotterdam market has a low influence on the Brazilian market of Paranaguá (13.56 %). This finding contrasts with previous studies, such as [3,16], which have empirically demonstrated a strong influence of Rotterdam on the Paranaguá spot, suggesting that price formation relies on the demand side (Rotterdam) rather than the supply (Brazilian). However [30], research was based on a much earlier period, during which market conditions might have been different. Furthermore [3], used a different methodology, which might explain the divergence in results.

However, Table 2 shows a high influence of Rotterdam on Chicago (18.5 %), which contradicts the findings of previous studies [3] that have demonstrated a lack of bidirectional-causality between Rotterdam and Chicago Futures [3]. results may seem counterintuitive, as they suggest a lack of causality between two of the most important markets in the international soybean market, which goes against the Law of One Price (LOOP). The influence of Chicago on Rotterdam is slightly higher (20 %) than vice versa (18.5 %). This can be easily explained by the fact that Chicago is a price leader and the largest market in the world [16] However, the influence of Rotterdam on Chicago is still high and significant. This can be explained by Chicago being a futures market and taking into account market futures expectations, harvest futures, stocks, and future demand (e.g., consumption in developing countries and EU union futures demand), US monetary policy, financial speculation, and energy commodities, etc. [31].

For other markets, the influence of Rotterdam is relatively low and limited (Paranaguá Spot 13.56 %, Rosario Spot 12.99 %, Rosario Futures 12.95 %, Dalian Futures 4.96 %, and China Spot 4.39 %). This follows a pattern where the influence decreases with the degree of market intervention or increases with market freedom, which is consistent with economic theory and market integration. The received spillovers “FROM” other markets (73 %) are higher than the spillover transmitted “TO” others (67.37 %), making Rotterdam market a net receiver (5.63 %). This means that Rotterdam is no longer a price leader and has lost its prominence in the international soybean market. This result goes against previous empirical research from Refs. [3,16] that highlighted the importance of Rotterdam as a price maker.

The Chicago market has a high influence on the Paranaguá spot market (19.11 %), and as previously mentioned, Paranaguá also has a similar influence on the Chicago market (19.3 %). This demonstrates that the causality between both Chicago and Paranaguá is bidirectional and suggests that Paranaguá is moving toward price leadership. The Chicago market also has a high influence on Rotterdam (20 %) due to its leadership status, which is an intuitive finding. The influence of Chicago significantly decreases for the Argentinian (Rosario futures 15.69 % and Rosario Spot 9.89 %) and Chinese markets (Dalian Futures 7.55 % and China Spot 10.59 %), following the previously mentioned pattern and likely associated with government intervention and market freedom.

The observation to consider is that Chicago seems to exert more influence on the China Spot market, known for structural intervention, as opposed to the Dalian futures market, which enjoys a higher degree of market freedom. The explanation relies on the fact that the Chinese government uses Chicago prices as a reference for fixing the domestic market [32], while the Dalian market relies more on the market price discovery process and futures expectations, being less influenced by Chicago. [33], using IRF and FEVD, stated that the information transferred from Chicago to Dalian was very similar in magnitude (CBOT attributed to DCE 0.1 %–5 % and DCE attributed to CBOT 5 %–25 %). This follows the results of this research, where the net transmitted from Chicago to Dalian was 7.5 % and from Dalian to Chicago was around 6 %. However [33], Han et al. (2013) stated that the facts reveal the key role of Dalian in global soybean price discovery. However, the evidence supports that the influence of Dalian, despite being bidirectional and of the same magnitude, is quite low in comparison to other markets that tend to influence Chicago. For instance, Paranaguá (19.3 %), Rosario futures (17.5 %), and Rotterdam (18.51 %) spillover is approximately three times higher than Dalian. In contrast to previous research, Chicago futures transmit slightly fewer shocks (15.69 %) than shocks received from Rosario Futures (17.54 %), indicating bidirectional causality in favour of Rosario Futures. These findings contradict previous empirical evidence suggesting a change in leadership. Finally, Chicago positions itself as a net transmitter of shocks, with a total spillover “TO” of 82.88 % against received shocks “FROM” 71.38 %, with a net transmission of 11.5 %.

Rosario futures have the highest connectedness and influence on Rosario spot at 25.04 %, confirming previous research from Ref. [3], which showed that Rosario futures can be associated with approximately half (47 %) of Rosario spot price decreases and cause Rosario spot. The influence fades to 16.97 % in the other direction. Rosario futures also show a higher degree of influence on Rotterdam than the other way around, which goes against what previous researchers have suggested [10]. had suggested that price formation relies on the demand side (Rotterdam) and expected this market to hold a higher level of influence. As previously mentioned, the influence of Rosario futures in Chicago is higher than vice versa (17.5 % vs. 15.7 %). This positions Rosario in a position of price leadership. However, Rosario Futures has marginal influence on Dalian futures (5.5 %) and China spot (4.4 %). Rosario Future is mostly influenced by itself (28.97 %) followed by Rosario Spot (16.97 %), and at the same time, it transmits shocks or spillover to Rosario Spot at a higher degree (25 %). Rosario Futures and Spot have a complementary relationship, as previously explained by Ref. [3]. Finally, Rosario Futures can be considered a net transmitter of shocks, transmitting 84.95 % (TO) and receiving 71.03 % (FROM), leaving a positive spillover of 14 %. The question that arises is why Rosario Futures shows a high degree of influence on other markets while the Argentina Spot market is highly suffocated by government intervention.

The influence of Rosario Spot is considerably low for almost all-time series (9.9 % for Rotterdam, 7.9 % for Chicago futures, 7.68 % for Paranaguá, 2 % for Dalian Futures, and 1.2 % for China Spot). However, the influence of Rosario Spot on Rosario Futures is considerably high at 16 %, as expected from complementary markets. Rosario Spot is highly influenced by itself (31.48 %) and as previously mentioned, influenced by Rosario Futures (25 %), followed by Rotterdam (12.99 %), Paranaguá (11.8 %), Chicago (9.9 %), China Spot (4.8 %), and Dalian Futures (3.98 %). Rosario Spot receives “FROM” the studied markets 68.52 % and transmits “TO” other markets 45.72 %. Therefore, it is possible to affirm that Rosario Spot is a net receiver (22.8 %). This raises the question of why the influence of Rosario Spot is so low in comparison with Rosario Futures. The first thing to understand is that Rosario Spot is a market where Argentine farmers sell their production, and this market has a degree of market intervention. There is an export tariff called “retentions” of around 33 %–35 %, which may prevent domestic and international price convergence, partially dislocating the price and insulating the market [34]. This might explain the low connectedness, relatively low “FROM” and “TO” other market influence found in this market. Moreover, currency control and restrictions might further disconnect the market from international markets.

Dalian Futures market tends to be marginally affected by international markets and seems to be affected primarily by itself (63 %), and marginally affected by Paranaguá (8.33 %), China (8.6 %), Chicago (7.6 %), and Rosario Futures (5.5 %). This means that there is no clear evidence of a complementary relationship between the Chinese futures market and the spot market. This can happen due to a high degree of market intervention that disconnects both markets, as is the case with China Spot intervention [32]. Therefore, this might explain the marginal influence of Dalian Futures on China (6.98 %), but still, the influence of China's domestic market on Dalian is low; however, in comparison to other markets, it is quite high in relative terms. China Spot is the second most important market that influences Dalian. However, it is challenging to explain the lack of influence of Dalian Futures on the international soybean market, as it is the second-largest market globally in terms of value [35]. The influence is marginal, and Dalian is classified as a net receiver −1.93 % (TO 34.78 %, FROM 36.71 %).

Finally, China seems to mainly influence itself (63.29 %), presenting marginal influence on other markets, and only having relative influence on Dalian Futures (8.25 %). In relative terms, it is modestly influenced by other markets, being affected by Chicago Futures (10.59 %), Paranaguá (9.19 %), Dalian Futures (6.98 %), and Rosario Futures (4.41 %). This lack of connectedness can be explained by the degree of market intervention presented in this market [2,6]. already pointed out the difficulty in finding cointegration within the market with international markets and the lack of significant regressors in the VECM, finding only a significant long-term regressor in Chicago Futures. This suggests that Chinese policymakers use Chicago prices as references to fix prices [32]. Potentially, Chinese policymakers are using Paranaguá prices as references as well. Finally, China's spot market behaves as net receivers −12.6 % (TO 24 %, FROM 36.7 %)

**Chart 6:**
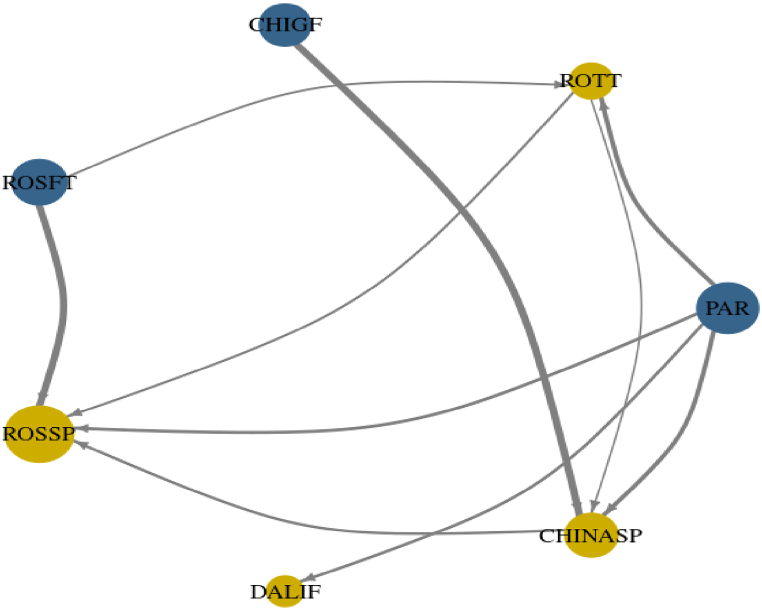
Network plot.

Chart 6 is a network plot that shows the net transmitters in blue and net receivers in yellow. The size of the circles represents the relative importance of the transmitter and receiver markets. This chart illustrates the dynamics of the international market, and even indirectly, causality. Previous researchers have demonstrated the price leadership of Chicago and Rotterdam. In contrast, the results show the ascension of Paranaguá and Rosario futures market as a price leader, affecting all transmitter markets to all receiver markets in conjunction with Chicago. The results clearly show that Chicago futures have lost international price leadership, in favour of a triumvirate with Rotterdam and Paranaguá, which goes against the results of previous empirical research from Ref. [3].

The results suggest a gradual fading of price leadership or a shift in price leadership for the researched period. This decline in Chicago's leadership is an interesting interpretation. This decay seems to be paired with the ascension of Paranaguá and Rosario Futures, as previously mentioned. The decline interpretation can be seen as a strong statement, as different authors have arrived at different results using different methodologies. This raises questions about the suitability and robustness of these different methodologies. Chicago remains a net transmitter, but its influence is limited to China. The China spot market uses Chicago prices as a reference price for fixing their prices [32]. Therefore, as long as Chinese policymakers believe that Chicago represents the international price and use this price as a reference, Chicago's influence will remain. The results showed that the Rotterdam market should be classified as a net receiver, being affected by Paranaguá and Rosario futures. This finding contrasts with [2,7], who found that in terms of price transmission, causality works the other way around (from Rotterdam to Paranaguá and Rosario futures). Despite being classified as a net receiver, Rotterdam also transmits shocks to Rosario spot and China spot, which is consistent with previous research results by Ref. [3].

Rosario futures appear as a net transmitter. As previously mentioned, this market transmits to Rotterdam and Rosario spot. The relationship of futures affecting the spot market (from Rosario futures to Rosario spot) can be explained by futures expectations affecting actual prices, such as next harvesting yield expectations, expected demand increases or decreases, global recession, or global growth. All this information is compressed in the futures price and affects the spot market [25,29]. The Rosario spot market shows itself as a net receiver, affected by China spot, Paranaguá, Rotterdam, and as previously mentioned. The China spot market shows itself as a net receiver, being affected by Chicago, Rotterdam, and Paranaguá, with the most significant impact coming from Chicago.

This result aligns with [15], who demonstrated the Granger causality from Chicago, Rotterdam, and Paranaguá to China and developed a global VECM (using the same markets) that revealed the only statistically significant regressor remaining in the long-term equation was from Chicago, signifying its importance for the China spot market. Finally, Dalian futures appears as a highly insulated market, being affected only by Paranaguá. In contrast [36], found that Dalian was a satellite market subordinate to Chicago. This research suggests that Dalian might be a subordinate market under the influence of Paranaguá. However, it is worth noting that [36] was conducted prior to the rise of Brazil and Argentina in the international market.

**Chart 7:**
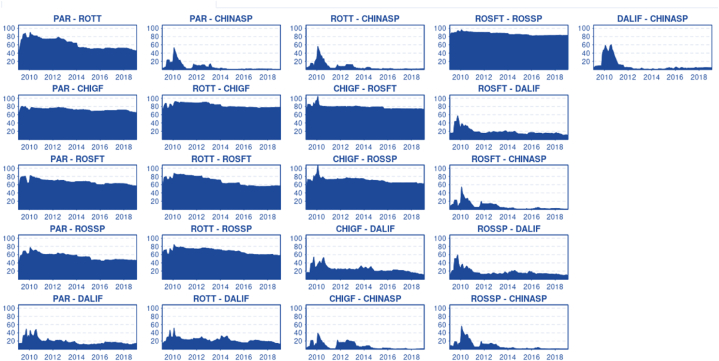
Dynamic pairwise connectedness.

In Chart 7, we can observe the pairwise connectedness. A higher level of cointegration and market efficiency is reflected in stable pairs, as prices move together, and shocks are uniformly transmitted. The highest connectedness and most stable pair are Rosario Futures and Rosario Spot, demonstrating clear cointegration and potentially efficient price transmission. This finding is expected since both markets are complementary. Paranaguá and Chicago also form a pair, excluding the period from 2009 to mid-2010, which distorts the results. This outcome is anticipated due to the strong cointegration among these markets and the efficiency represented by fast and symmetrical price transmission (Error correction term: 30 % per month) [3,15]. Excluding the period with diverse exogenous shocks causing large price swings [26] (2009-mid 2010), pairwise connectedness tends to be relatively stable, at least for highly cointegrated markets (Rotterdam-Chicago, Chicago-Rosario Futures, Rosario Futures-Rosario Spot, Rosario Futures).

For the Chinese spot market, the pairwise connectedness tends to be variable and highly unstable, even for the pair China Spot-Dalian Futures. Dalian Futures has a higher connectedness index compared to China Spot, but not as high as the western markets. From mid-2009 to 2011, the dynamic pairwise connectedness for the Chinese domestic market peaks with almost all markets. This period suggests that the China spot market might have been affected by a combination of exogenous supply shocks that the world experienced during that time, or that Chinese droughts may have pushed prices up, affecting domestic demand and increasing soybean imports. Dalian's dynamic pairwise connectedness appears to fade over time with highly cointegrated markets such as Chicago, Rosario Futures, Paranaguá, and Rotterdam, indicating further future market isolation. As mentioned earlier, connectedness is an analogous concept to cointegration in a dynamic sense, and it seems that market cointegration is one of the main components affecting the pairwise connectedness index. The pairwise connectedness of Paranaguá-Rotterdam tends to be unstable and decrease over time. To a lesser extent, the same occurs with Rotterdam-Rosario Futures and Rotterdam-Rosario Spot. This can be interpreted as the stagnant demand for soybeans from the EU compared to other markets, causing Rotterdam to lose pairwise connectedness with the main markets, Paranaguá and Chicago. In other words, Rotterdam's leadership and importance in terms of prices and demand are diminishing.

**Chart 8:**
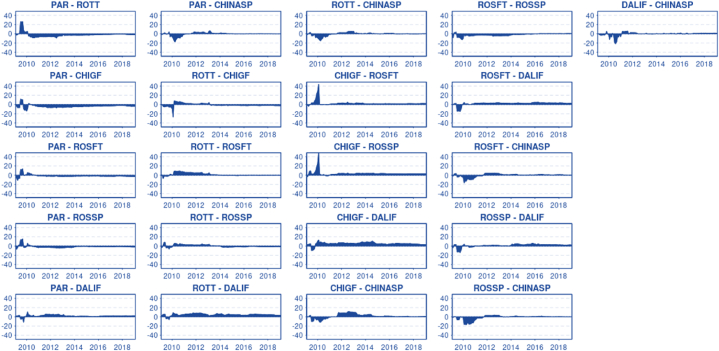
Net pairwise directional connectedness.

Chart 8 displays the net pairwise directional connectedness, allowing us to appreciate the spillover direction in pairwise markets [26]. correlated several events that may have caused price shocks from the beginning of 2010 to mid-2011 and illustrated how these events might have pushed up prices and generated long swings in the market (Chart 2). Interestingly, the diverse nature of these events made it difficult to pinpoint the exact nature of the shocks and the weighted causality. No empirical work has proven causation for the different events related to market instability. However, using pairwise connectedness, it is possible to understand the origin of the shocks. A common pattern in the different pairwise combinations consistently show the Asian markets as responsible for the spillover to other markets during the previously mentioned instability period. After that period, the Chinese market shifted from being net transmitters of shocks to being net receivers. The influence of the Chinese market on the international market could be attributed to a combination of Chinese importers aggressively purchasing soybeans to secure a six-month supply and imminent dryness in China, which could depress soybean production or generate future expectations of a shortage, or directly shift the supply curve to the left. This occurred in both futures and spot markets. It is possible to observe that during the disturbed period, the pairwise connected spillover between Dalian and China shifted a couple of times, but on average, the China spot market had the most significant influence. In addition, excluding the Asian market, it seems that for the disturbed period, Paranaguá reacted faster than other western markets, likely adjusting the price first and influencing Rotterdam. Chicago Futures also responded faster, alternating between receiving and transmitting shocks with Paranaguá.

The net pairwise directional connectedness could be useful in understanding causality from one market to another over time. Incorporating additional analyses, such as Granger causality tests, could enhance our comprehension of the underlying dynamics. If we analyze the net pairwise directional connectedness for each case, excluding the previously mentioned disturbed period, we can draw the following conclusions:

The Paranaguá-Rotterdam pair shows a fading influence of Rotterdam over time, where previous work by Ref. [3] shows that Rotterdam Granger-causes Paranaguá. The Paranaguá-China Spot pair clearly positions Paranaguá as a net transmitter, influencing China Spot. The Rosario Futures-Rosario Spot pair demonstrates the consistent influence of Rosario Futures over Rosario Spot over time, in line with the Granger causality results from Ref. [3]. Similarly, in line with the previously mentioned research, Dalian Futures have had a more significant influence on China over time.

The Paranaguá-Chicago Futures pair shows that over time, Chicago has had more influence on Paranaguá; however, that influence seems to be decreasing over time. Again, this is in line with the previous researchers' Granger causality tests. The Rotterdam-Chicago pair, according to the previous researchers, had no causality between them. The pairwise directional connectedness showed an abrupt change in causality at some point in mid-2013; from then on, the influence of Chicago over Rotterdam has been slowly and consistently growing over time. This suggests that by the end of the studied period, Chicago probably Granger-caused Paranaguá.

Regarding the Chicago Futures-Rosario Futures pair, the previous researchers stated that there was causality between both markets. The pairwise net connectedness shows that Chicago slightly influences Rosario Futures; however, it is not clear if that influence is decreasing over time. In the case of Rosario Futures and Dalian Futures, the empirical evidence from Ref. [3] supports that there is no causal relationship between both markets. In contrast, the directional pairwise connectedness suggests Rosario Futures clearly influence Dalian Futures, transmitting shocks to the Asian market. This also occurs with Paranaguá and Rosario, where the previous researchers empirically demonstrated no causation between both markets; the pairwise directional connectedness shows that Rosario Futures might influence Paranaguá. The explanation for this relies on the nature of both markets: while futures markets depend on future expectations, future demand, yields from upcoming harvests, exogenous events, and pure speculation, spot markets rely on actual demand and supply, and future prices can prompt suppliers to adapt by increasing or decreasing production according to price expectations. Therefore, it is understandable that Rosario Futures and Chicago Futures affect Paranaguá. However, the opposite happens with Paranaguá and Dalian, and these results are in line with the previously mentioned researchers.

The Rotterdam-Rosario Futures net pairwise directional connectedness shows that, for the first part of the period, Rotterdam had a more significant influence on Rosario Futures. At some point in 2013, this influence ended, and there was a neutral balance between both markets, indicating no causality between them. This finding is in line with the previous researchers' conclusions.

The Chicago Futures-Rosario Spot net pairwise directional connectedness clearly demonstrates causality from Chicago Futures to Rosario Spot, which is consistent with the previous researchers' findings. Rosario Futures show a clear influence on China Spot after the turbulent period, again in line with [3]. The Rosario Spot-Paranaguá pair showed that Rosario Spot caused Paranaguá until 2014. The reason behind this could be a left shift in Argentina's market supply in 2014, followed by changes in the export tariff regimen, which might have transmitted shocks to Paranaguá. The stability of Paranaguá does not make it a source of spillovers. After 2014, the balance between the pairs remains neutral, and no causality occurs between them. The previous researchers stated that Granger causality goes from Paranaguá to Rosario Futures.

The Rotterdam-Rosario Spot pair shows an effect going from Rotterdam to Rosario Spot. From 2014, there is a shift in causality from Rosario Spot to Rotterdam. However, the effect tends to be rather marginal, between 1 and 20 %. The previous researchers empirically demonstrated that causality goes from Rotterdam to Rosario. The Chicago Futures-Dalian Futures net pairwise directional connectedness clearly shows a significant influence of Chicago on Dalian, which contradicts and aligns with [3]. The pairs with Dalian or Rosario Spot - Paranaguá or Rotterdam all exhibit the same result as Chicago-Dalian: a clear effect from those markets on Dalian Futures, consistent with findings from the previously mentioned researchers. Chicago Futures-China Spot initially shows a significant effect going from Chicago to China that decreases over time and tends to be neutral in 2016. This can be explained by active market intervention to reduce international spillover into the domestic market. To a lesser degree, the same pattern can be observed in the Rosario Spot-China Spot pair. According to Ref. [3], causality goes from Chicago and Rosario Spot to China Spot. Granger Causality results for the same series and period can be appreciated in Table 3.

**Table 3:** Granger Causality test [3].

Granger Causality Test with One Lag							
	LNCHIGF	LNCHINASP	LNDALIF	LNPARANAGUA	LNROSFT	LNROSSP	LNROTTERDAM
LNCHIGF	X	↑	↑	↑	∂	↑	∂
LNCHINASP	←	X	←	←	←	←	←
LNDALIF	←	↑	X	←	∂	←	←
LNPARANAGUA	←	↑	↑	X	∂	↑	←
LNROSFT	∂	↑	∂	∂	X	↑	∂
LNROSSP	←	↑	↑	←	∂	X	←
LNROTTERDAM	*∂*	↑	↑	↑	∂	↑	X

The direction of the arrows (←, ↑) represents the direction of the causality. ∂ represents no causality affectation.

## 4 Discussion and policy implications

Empirical evidence suggests that the degree of market freedom plays a fundamental role in the efficient transmission of market signals and faster reactions to international demand. The expansion of soybean production in Brazil [27] and Argentina [28] serves as a perfect example. While the Argentinian government imposed several restrictions, including export tariffs and fixed exports, the Brazilian government did not interfere with soybean expansion in the country. As a result, Argentina struggled to sustain soybean expansion and missed out on the economic growth it could have generated. Moreover, this intervention led to a significant exodus of farmers and capital to neighbouring countries such as Paraguay and Uruguay [37], where the conditions for farming and exporting production were more favourable. In contrast, Brazil capitalized on the economic benefits of soybean expansion, strengthened its domestic market, and competed with the United States in soybean exports. The prolonged market freedom and economic stability in Brazil allowed for the development of the domestic market and the expansion of soybean crops across the fertile soils of the Brazilian states of Mato Grosso, Paraná, and Rio Grande do Sul, achieving 36.95 million hectares in the 2019/2020 season [38]. In the same season, Argentina's soybean area was estimated at 14.92 million hectares [39]. This development eventually paved the way for Brazil to become the price leader and a worldwide reference in terms of soybean production.

Many empirical research studies have shown that the US has continued to hold price leadership in this market [3,16] but this research diverges. However, the empirical evidence still indicates that the importance of the US remains crucial. The US has experienced a high degree of market freedom and a well-developed market for a long time, with the Chicago Board of Trade being one of the oldest futures markets in the world, inaugurated in 1848 and first started operating with soybean contracts in 1984. The question that arises is why the US leadership is declining and being overtaken by Brazil. While it may be too early to suggest that this is happening, there are three main factors that might have affected the price leadership. Firstly, the US-China trade war has actively redirected trade flows, making Brazil the primary supplier to China for a period. Secondly, soybean production in Brazil continues to grow, surpassing the US in 2019 and projected to produce almost 24 % more than the US by 2023 [40].

Finally, the Chinese government's intervention in the domestic market with various price support policies has isolated the market from international market fluctuations. Previous researchers have struggled to find cointegration [15] or found cointegration after correcting the series for structural breaks and using different methodologies [3], suggesting the fading of the cointegration vector due to government intervention. This is in line with previous research, despite differing methodologies, as the Chinese domestic market exhibited the lowest TCI of the time series for both the total connected index and dynamic pairwise connectedness. However, before 2012, both indicators showed noticeably higher spill-over, suggesting a period of less government intervention and higher market integration. After that, the TCI index decreased marginally, indicating a more isolated market.

[3] found that the most significant market for China's domestic market was Chicago Futures, which aligns with this research, showing that China's domestic market is affected by the main markets of Chicago, Rotterdam, and Paranaguá, but Chicago has the most significant impact on the Chinese domestic market. The previously mentioned research suggests that this might happen because Chinese policymakers use Chicago prices as references to fix domestic prices. Chinese policymakers may need to reevaluate their reference market and use Paranaguá as the new price reference. It is well known that any price-fixation policy will distort the market, but if your price-fixation equation incorporates a term with a price that is no longer an international reference, the market distortion will be higher.

The Chinese government, through the 14th five-year plan (Ministry of Agriculture and Rural Affairs), is seeking to boost soybean production from 16.4 million tonnes in 2021 to 23 million tonnes by 2025, an increase of 40 % to ensure food security [29]. However, these attempts at self-sufficiency may fall short, as 23 million tons of soybeans represent only 22.5 % of total imports. China's new policy aims to increase subsidies for soybean farmers, extend credit lines for farms, and offer insurance to cover costs and ensure incomes. These incentives aim to engage Chinese farmers from the North and Southeast and the lower and middle reaches of the Yangtze River to participate in a pilot program where soybeans and corn are grown alongside each other. In addition, the implementation of new technology, such as innovative cultivation methods, machinery, and seeds, provides a competitive edge to increase yields [41]. These policies will increase domestic soybean production, aiming to substitute soybean imports with domestic production. This will lead to a drop in demand for international soybeans, causing a fall in prices in the international market, which will have a knock-on effect on Chinese domestic prices, decreasing the price. These attempts by the government to achieve food security might empower China's domestic and future markets if this policy follows a price liberalization policy, potentially integrating market cointegration and boosting the efficiency of price transmission.

The decline of Rotterdam as a price leader and price maker is evident, with the results showing Rotterdam as a net receiver, influenced by Chicago Futures, Paranaguá, and Rosario Futures. However, the net balance is slightly negative, meaning that Rotterdam still serves as a source of shocks and spillovers to other markets, particularly Chicago and Rosario Spot. The question that arises is how Rotterdam's influence has faded and how this important market has transitioned from being a price leader to a price taker, influenced by other markets. First, it is essential to understand that Rotterdam represents the European Union and the demand side; the role of the European Union as a major importer of soybeans has eroded over the last 20 years, as the demand for soybeans from China has consistently grown, while the European Union's demand has remained relatively stagnant. Over the years, China has overtaken the European Union in terms of demand, accounting for 65 % of international soybean imports. This process has weakened Rotterdam's price leadership.

Argentina's current and future role in the international market is clouded by a high level of uncertainty. The current government has imposed strict trade restrictions, such as export tariffs and currency manipulation, followed by regional droughts that have severely impacted yield expectations. The future of Argentina's soybean farmers seems to be highly compromised, at least in the domestic market (Spot market). The presidential elections in October 2023 may bring economic liberalization, lifting currency restrictions and export tariffs, potentially increasing Argentina's market efficiency and integration, and unlocking its full potential in terms of soybean exports. Otherwise, Argentina's future role in the international soybean market may be further jeopardized. In the meantime, despite the high level of intervention in the domestic market, the Argentine Futures market has positioned itself as a price leader, price maker, and net transmitter of shocks and spillovers, influencing major markets such as Paranaguá, Chicago, and Rotterdam.

The reign of Chicago and Rotterdam as canters of price formation seems to have come to an end. Rotterdam has fallen to a secondary role in the international soybean market, while Paranaguá (Brazil) and Rosario Futures (Argentina) have risen to lead the international market alongside Chicago. This new triumvirate maintains price leadership and competes for market share. The Chinese government has adopted an active strategy to ensure food security by increasing domestic production, but this seems to be unattainable in the short to medium term. China has failed to capitalize on its position as the world's leading soybean importer and translate this into market power, thereby empowering the futures and domestic markets. Both markets remain relatively insulated and disconnected from the mainstream, unable to influence international prices and acting as net receivers. Dalian Futures, the world's second most important agricultural futures market, is still far behind in price leadership and market efficiency.

This innovative methodology has allowed us to gain a deeper understanding of the international soybean market from a time-varying perspective. One of the key findings is that causality is not static over time and is generally bidirectional, with shifts in causality occurring over time. Shocks can be transmitted from satellite markets to mainstream markets, and the connectedness index clearly demonstrates that these shocks and spillovers can originate from any part of the international market and be transmitted horizontally. The efficiency of price transmission may play a fundamental role in conveying these spillovers. The connectedness approach methodology makes it easier to correlate exogenous shocks with spillovers in the market, allowing for a more accessible understanding of the origin of exogenous shocks and tracking them across the market's spatial dimensions.

## 5 Conclusion

The dynamic connectedness methodology outlined in Ref. [1], based on a Time-varying Parameter Vector Autoregressive (TVP-VAR) model and the connectedness index [2], has proven to be a game-changer in understanding market integration and price transmission. It introduces the concept of dynamic cointegration and time-varying causality, which better aligns with market processes where shocks, price changes, and shifts in demand and supply occur across the international market in multiple directions. This research focuses on understanding the international soybean market by studying the US market (Chicago Futures), Rotterdam representing the European soybean market, Paranaguá market on behalf of Brazil, Argentina represented by Rosario Futures and Spot, and finally the Chinese domestic spot market and Dalian futures on behalf of China. The study period covers approximately ten years from September 2009 to May 2019. The results suggest that the soybean market is highly mature and capable of circumventing exogenous shocks, at least for the past seven years. The dynamic connectedness index revealed a highly connected and developed market (67 % average connectedness). The research found a higher degree of connectedness in the western market (Chicago Futures, Rotterdam, Paranaguá, and Rosario Futures and Spot). However, the connectedness between the Western and Eastern markets (China Spot and Dalian) was quite low, indicating market isolation. Furthermore, the pairwise connectedness index between Dalian Futures and China Spot market appeared to be quite low, and the GFEVD demonstrated that both China and Dalian are net receivers, being influenced to varying degrees by most western markets and denoting market isolation. The pairwise net directional connectedness was able to trace the origin of market disturbances that occurred between 2009 and 2011, as explained by Ref. [26]. The test indicated the origin of the disturbance in China, attributed to dryness and changes in import strategies that shocked the international market and were transmitted from the Eastern to Western markets, with China as the epicentre. The net pairwise connectedness also showed that causality between the markets is not static and is rather dynamic, changing over time. The results reveal the ascension of Paranaguá and Rotterdam as price leaders, affecting most markets to varying degrees. Rotterdam's leadership seems to be fading over time, likely due to stagnant demand and a lack of growth in this market over recent years. Chicago remains one of the most important markets, exerting a strong influence on most markets, but sharing price leadership with Paranaguá and Rosario in a dynamic triumvirate of net shock transmitters.

### 5.1 Suggestion for further research

The TVP-VAR connectedness index methodology is an innovative technique that offers opportunities for additional investigation into price transmission and market integration. It would be insightful to examine the cross-price transmission occurring between the international soybean market, other agricultural commodities, and energy commodities such as crude oil, natural gas, and various fertilizer prices. Analysing the connectedness among these commodities and identifying the causality in terms of net transmitters and net receivers could prove valuable. Another suggestion is to refine the granularity of the data and employ the QVAR models and connectedness index methodology to study more recent geopolitical events, such as the Russian-Ukrainian conflict. By doing so, researchers can assess the impact of the conflict on agricultural commodities and trace the spillover effects transmitted across different energy and agricultural commodities.

## Data availability statement

Data will be made available on request.

## CRediT authorship contribution statement

**Gustavo María Barboza Martignone:** Writing – original draft, Methodology, Investigation, Conceptualization. **Bikramaditya Ghosh:** Software, Methodology, Formal analysis, Data curation. **Karl Behrendt:** Writing – original draft, Supervision. **Dimitrios Paparas:** Writing – original draft, Supervision, Investigation.

## Declaration of generative AI and AI-assisted technologies in the writing process

During the preparation of this work the author(s) used ChatGPT 4 to improve the English. After using this tool/service, the author(s) reviewed and edited the content as needed and take(s) full responsibility for the content of the publication.

## Declaration of competing interest

The authors declare the following financial interests/personal relationships which may be considered as potential competing interests:Gustavo Barboza Martignone reports writing assistance was provided by Harper Adams University College Department of Land Farm and Agribusiness Management.
